# Trial-Adjusted Versus Generic Simulated Comparator Trial (SCT) Settings for Selection Bias Appraisal Using the I2 Test

**DOI:** 10.7759/cureus.71668

**Published:** 2024-10-16

**Authors:** Steffen Mickenautsch, Veerasamy Yengopal

**Affiliations:** 1 Faculty of Dentistry, University of the Western Cape, Cape Town, ZAF; 2 Community Dentistry, University of the Witwatersrand, Johannesburg, ZAF

**Keywords:** bias identification, randomized clinical trial, review of clinical trials, selection bias, systematic review and meta analysis

## Abstract

Aim: The aim was to test two null hypotheses: that I^2^ testing with trial-adjusted simulated comparator trial (SCT) settings does not change the odds of identifying selection bias in clinical trials compared to I^2^ testing with generic SCT settings, and that I^2^ testing with trial-adjusted SCT settings does not change the odds of identifying selection bias in smaller trials (with sample size (n) = 100-199 per treatment group) compared to larger trials (n > 200 per group).

Methods: Baseline data from 67 randomized controlled trials previously tested for selection bias using the I^2^ test with generic SCT settings were extracted. The generic settings were: SCT sample size N_SCT_ = 200 (100 for each of Groups A and B), minimum-maximum range of random values (R_SCT_) = 67 (minimum = 18, maximum = 85), number of generated SCTs used in all meta-analyses (SCT_N_) = 2. The trials were re-tested with trial-adjusted SCT settings. Additionally, the SCT sample sizes were further increased stepwise to N_SCT_ = 400, 800, and 1200, and the resulting I^2^ point estimates were recorded. Positive test results (I^2^ > 0%) were assigned a score of 1, while negative test results (I^2^ = 0%) were assigned a score of 0. From the resulting 0 and 1 scores of both types of SCT settings, odds ratios (ORs) with 95% confidence intervals (CIs) and p-values were computed. The alpha level was set at 5%.

Results: The original I^2^ testing with generic SCT settings yielded four positive and 63 negative results. In contrast, testing with trial-adjusted SCT settings of the same trials revealed 13 positive and 54 negative results (OR: 3.79; 95% CI: 1.17 - 12.32; p = 0.03). When the SCT sample size was increased with trial-adjusted SCT settings, the number of positive results rose from 13 to 16 (OR: 1.30; 95% CI: 0.57 - 2.98; p = 0.53). Consequently, only the first null hypothesis was rejected.

Conclusion: I^2^ testing with trial-adjusted SCT settings increased the odds of identifying selection bias in clinical trials and did not significantly alter the odds in smaller trials with fewer than 200 patients per intervention group.

## Introduction

Selection bias distorts the true effect estimate in randomized control trials (RCTs) when patients with characteristics conducive to a successful outcome of one particular treatment over another are not allocated randomly to treatment groups [1,2]. This non-random allocation creates imbalances between the baseline variables of these groups that can be detected in the form of in-between study heterogeneity using baseline variable meta-analysis, where baseline variable values from such trials are statistically pooled with those from non-biased RCTs [3,4]. Any between-study heterogeneity of baseline variables (such as age, weight, height, etc. per treatment group) can only occur either due to play of chance or due to some problems in the randomization process [5]. The I^2^ statistic is commonly used in meta-analyses to indicate between-study heterogeneity beyond chance, denoted by an I^2^ point estimate > 0% [6,7]. Against this background, Hicks et al. (2018) proposed a test method to identify selection bias in outcomes meta-analyses [3].

Mickenautsch and Yengopal (2024) applied the same principles [1-3,5] to detect selection bias in single trials [8]. However, in this method, instead of pooling several RCTs according to the method by Hicks et al. [3], the baseline variable values of the trial to be tested are pooled together with simulated values that are specifically generated to not contain any baseline imbalances. Such lack of baseline imbalance creates a ‘perfect world’ scenario where no selection bias exists and is reflected by an I^2^ = 0% value in a fixed-effect meta-analysis of so-called ‘simulated comparator trials (SCTs)’. 

At least two SCTs are generated, each consisting of three data columns in MS Excel (Microsoft Corporation, Redmond, Washington, United States): an ascending list of integers (1,2,3, … ), serving for data point identification (Column 1), a random ‘A, B’ allocation sequence (Column 2), and a list of, within a specified range (minimum-maximum value), randomly generated values (integer or decimals with random duplications) that are sorted in ascending order (Column 3). From each of these SCTs, the mean value (with standard deviation (SD)) for Groups A and B are calculated and entered together with the baseline values of the trial to be tested into a fixed-effect meta-analysis [8].

During the generation of SCTs, three parameters are set: the total number of data points (SCT sample size, N_SCT_), minimum-maximum range of random values (R_SCT_), and the number of SCTs (SCT_N_) to be used in the meta-analysis. It has been established that each of these parameters affects the test’s sensitivity for indicating a positive result (I^2^ > 0%). Accordingly, it is recommended to set the parameters at the following levels: SCT_N_ = 2 and N_SCT_ and R_SCT_ in line with that of the baseline variable values reported in the test trial, provided the test trial has a sample size of at least n = 100 per intervention group. For smaller trials (n < 100 per group), the sample size of all SCTs should be set at N_SCT_ = 200 (100 for each of Groups A and B) [9]. Such ‘trial-adjusted’ settings may differ in their ability to identify true positive trials with selection bias (I^2^ > 0%) to that of ‘generic’ (one-fits-all) settings, where all trials are tested with the same SCT parameter settings, i.e., without adjusting for the test trial's sample size and minimum-maximum range of baseline variable values. 

The aim of this study was to test the two null hypotheses: (H0-1) that I^2^ testing with trial-adjusted SCT settings does not change the odds of identifying selection bias in clinical trials from that of I^2^ testing with generic SCT settings and (H0-2) that I^2^ testing with trial-adjusted SCT settings does not change the odds of identifying selection bias in smaller trials (with sample size n = 100-199 per treatment group) in comparison to that of larger trials (n > 200 per group).

This manuscript has been made available online as a preprint in Authorea: www.authorea.com: Mickenautsch S, Yengopal V. Trial-adjusted versus generic simulated comparator trial (SCT) settings for selection bias appraisal using the I^2^ - test (Preprint). Authorea. 2024, 10.22541/au.172660483.33308453/v1.

## Materials and methods

The following analyzed baseline data were extracted from all 67 RCTs with sample sizes of 100-199 per treatment group, which were included in the trial cohort of a previous study (Appendices, Section 1) [10]: type of baseline variable, mean baseline variable values with SD, sample size (n) per test and control group, and I² test results (%) obtained using generic SCT settings. The generic SCT settings for the I^2^ tests were: N_SCT _= 200 (100 for each of Groups A and B), R_SCT_ = 67 (minimum = 18, maximum = 85), and SCT_N_ = 2 [10].

In order to test the null hypothesis (H0-1) that I^2^ testing with trial-adjusted SCT settings does not change the odds of identifying selection bias in clinical trials from that with generic SCT settings, testing was repeated for all 67 trials using trial-adjusted SCT settings. SCT settings were trial-adjusted for each trial, according to the following procedure.

The combined mean (SD) value for both treatment groups from the trial report (if not reported the value was estimated by following the steps presented in Appendices/Section 2) were extracted and a random A, B allocation sequence (Column 2) was generated using block randomization. The length of the sequence, in line with the combined trial samples size: n_1_ + n_2_, was extended in order to fit block size = 4 (Appendices / Section 3). The allocation sequence was generated with the Sealed Envelope online tool (Sealed Envelope Ltd., London, England, United Kingdom) [11].

Next, an ascending list of integers (1,2,3, … ) serving as data point ID (Column 1) with list length according to the length of the random sequence and a list of randomly selected values (integers or decimals with random duplications allowed) within a specified range (R_SCT_ / Minimum - Maximum value as per Step 1) were generated by use of an online random number generator [12]. The list of random values was sorted in ascending order (Column 3). In the next step, the random values of Column 3 were allocated according to Groups A and B allocation in Column 2 in MS Excel (Microsoft Corporation, Redmond, Washington, United States) (Appendices / Section 4).

The mean value (with SD) for Groups A and B for always two SCTs per clinical trial were calculated and were entered together with the sample size per group into a fixed effect meta-analysis (RevMan 5.0.24 software; The Cochrane Collaboration, London, England, United Kingdom). The analysis was conducted and the resulting 0% I^2^ point estimate was confirmed. Finally, the mean (SD) baseline values with the sample sizes of the test and the control group of the clinical trial to be tested were entered into the same meta-analysis; the analysis was repeated and the new I^2^ point estimate was recorded [8].

To test the null hypothesis (H0-2) that I^2^ testing with trial-adjusted SCT settings does not change the odds of identifying selection bias in smaller trials (with n = 100-199 per treatment group) compared to that of larger trials (n > 200 per group), the sample sizes for Groups A and B of both SCTs of each meta-analysis were stepwise increased to N_SCT_ = 400, 800, and 1200, and the resulting I^2^ point estimates were recorded.

If the repeated I^2^ point estimate was also 0%, the test result was considered negative, indicating no selection bias in the tested trial, and scored as "0". If the point estimate was I^2^ > 0%, the test result was considered positive, the tested trial was assumed to include selection bias and scored as “1”. The resulting 0 and 1 scores for both generic and trial-adjusted SCT settings were used to compute the odds ratio (OR) with 95% confidence interval (CI) (RevMan 5.0.24 software). The results from both types of SCT settings were then statistically compared. The significance level (alpha) was set at 5%.

## Results

**Figure 1:**
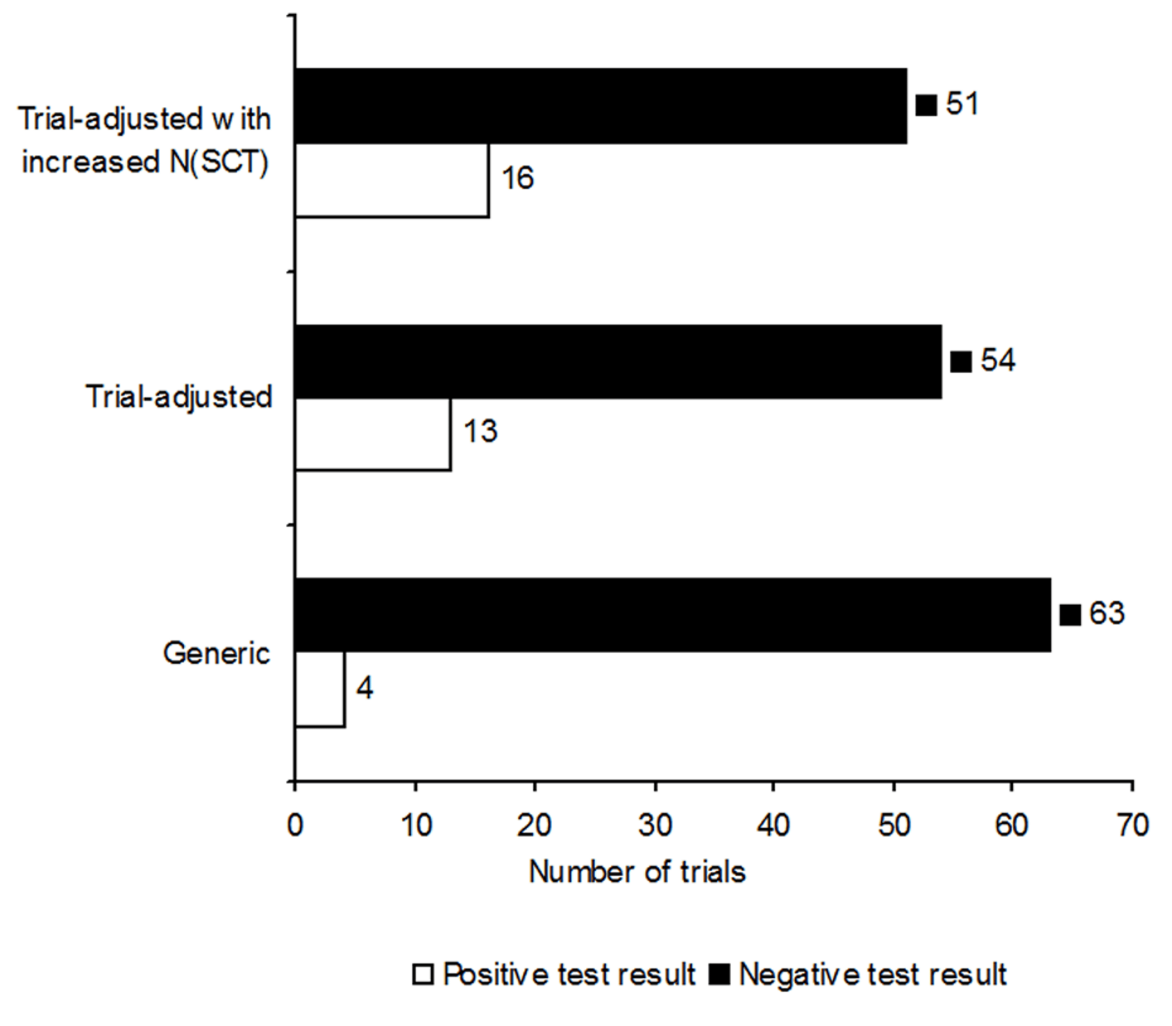
Test results per type of simulated comparator trial (SCT) settings

I^2^ testing with generic SCT settings of the 67 trials yielded four positive (I^2^ > 0%) and 63 negative (I^2^ = 0%) results. Testing with trial-adjusted SCT settings of the same trials yielded 13 positive and 54 negative results. Raising the SCT sample size when SCT settings were trial-adjusted increased the positive results from 13 to 16 (Figure 1, Appendices / Section 5).

**Figure 2:**
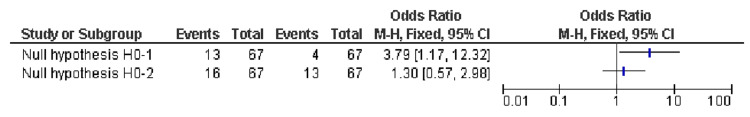
Null-hypotheses test results

Accordingly, I^2^ testing with trial-adjusted SCT settings significantly increased the odds 3.79 times above that for testing with generic settings (OR: 3.79; 95% CI: 1.17 - 12.32; p = 0.03; Figure 2). Hence, the null hypothesis (H0-1) was rejected.

Testing with trial-adjusted SCT settings for smaller trials initially produced three false negative results, but these were later corrected to positive results when the SCT sample size was increased. Of these, one false negative result was corrected at N_SCT_ = 400 and two at N_SCT_ = 800 when the SCT sample size was increased. However, the resulting difference was not statistically significant (OR: 1.30; 95% CI: 0.57 - 2.98; p = 0.53; Figure 2) and the null hypothesis (H0-2) was not rejected. The results of all conducted meta-analyses per trial are presented in Appendices / Section 1.

## Discussion

The aim of our study was to test the two null hypotheses: (H0-1) I^2^ testing with trial-adjusted SCT settings does not change the odds of identifying selection in clinical trials from that of I^2^ testing with generic SCT settings. (H0-2) I2 testing with trial-adjusted SCT settings does not change the odds of identifying selection bias risk in smaller trials (with sample size n = 100-199 per treatment group) in comparison to that of larger trials (n > 200 per group).

Only the first null hypothesis could be rejected. The odds of identifying trials with selection bias by use of I^2^ testing with trial-adjusted SCT settings were statistically significantly higher than with generic settings (OR: 3.79; 95% CI: 1.17 - 12.32; p = 0.03). The higher odds can be ascribed to the SCT sample size per group (mean = 264.06, SD: 50.67; Appendices / Section 4) of the former, which was more than twice as high than that for generic settings (100 per group for all trials); the R_SCT_ of the trial-adjusted SCT settings (mean = 75.22, SD: 156.62) was similar to that of the generic settings (R_SCT_ = 67). Because a higher SCT sample size is directly related to a lower 0/>0% threshold of the I^2^ point estimate [9], I^2^ testing with trial-adjusted SCT settings was more sensitive for identifying biased trials. Limiting the SCT sample size to that of the test RCT appeared to be sufficient, as further increases in the sample size up to N_SCT_ = 1200 only yielded a modest increase in positive test results, from 13 to 16 trials.

Furthermore, all trials identified with generic settings as biased were also identified with trial-adjusted SCT settings as being affected by selection bias (I^2^ > 0%). Hence, trial-adjusted SCT settings seem to maintain a high sensitivity for detecting selection bias, as they do not reduce the odds of a positive test result when bias is present. In summary, using trial-adjusted SCT settings for I^2^-based testing for selection bias in single RCTs appears not only to assure test reliability, due to independence from reviewer input, as well as SCT similarity with characteristics of the tested RCT [9], but also assures higher test accuracy by increasing the odds that a test is positive for an RCT when selection bias is present.

Limiting the SCT settings to that of the test RCT does not appear to significantly alter the odds of correctly identifying RCTs with high selection bias, particularly for trials with less than 200 patients per treatment group, compared to larger trials (as compared with larger trials, n > 200 / OR: 1.30; 95% CI: 0.57 - 2.98; p = 0.53). However, routinely increasing the N_SCT_ when a test result is negative (I^2^ = 0%) may assist in increasing the odds to correctly identify biased RCTs somewhat further. In light of this study’s findings, future research may include an update of the previous study by Mickenautsch and Yengopal (2024), which was based on generic SCT settings [10], using trial-adjusted SCT settings, instead.

Study limitations

The results of this study are limited by the characteristics of its used RCT sample, particularly its previously applied SCT sample size: N_SCT_ = 200 (100 per group) [10]. Especially the lower generic SCT sample size was the reason for the statistically significant results and subsequent rejection of null hypothesis H0-1. If the generic sample size had been N_SCT_ = 400, no significant difference in the odds may have been observed. The original generic SCT sample size of 100 per group was chosen based on meta-epidemiological evidence suggesting that trials with smaller sample sizes (< 100 per group) are at a higher risk of bias compared to trials with at least 100 subjects per intervention group [13-16]. Therefore, it may have offered an evidence-based and objective SCT setting for detecting selection bias in smaller trials. However, the lower SCT sample size in the generic setting substantially reduced test sensitivity, thereby increasing the risk of false negative results.

Furthermore, using all trials with sample sizes between n = 100 and 199 per group from the trial cohort of a previous study [10] has limited the sample size for this study to only 67. Therefore, the current results may be enhanced by future similar studies with larger trial numbers. Future research may also explore additional methods for generating reviewer-independent SCT settings that could potentially provide even higher sensitivity for single-trial I^2^-based selection bias testing.

## Conclusions

I^2^ testing with trial-adjusted SCT settings increased the odds of detecting selection bias in clinical trials and did not significantly alter the odds for smaller trials with less than 200 patients per intervention group. Therefore, using trial-adjusted SCT settings for I^2^-based testing for selection bias in single RCTs appears to offer a twofold advantage: (1) ensuring test reliability through independence from reviewer input and SCT similarity with the tested RCT's characteristics, and (2) enhancing test accuracy by increasing the likelihood of a positive test result when selection bias is present. Furthermore, routinely increasing the N_SCT_ when a test result is negative (I² = 0%) may provide additional assistance in boosting the odds of detection.

## Appendices

All data is fully available without restriction and can be freely downloaded via the link:

https://data.mendeley.com/datasets/wj28ysp99x/1
